# Analysis of Reported Health Care Use by Sexual Orientation Among Youth

**DOI:** 10.1001/jamanetworkopen.2021.24647

**Published:** 2021-10-29

**Authors:** Sari L. Reisner, Camila Mateo, Marc N. Elliott, Susan Tortolero, Susan L. Davies, Terri Lewis, Dennis Li, Mark Schuster

**Affiliations:** 1 Pediatrics, Boston Children's Hospital, Boston, Massachusetts; 2 Division of Endocrinology, Diabetes & Hypertension, Brigham and Women's Hospital, Boston, Massachusetts; 3 Department of Medicine, Harvard Medical School, Boston, Massachusetts; 4 Department of Epidemiology, Harvard T.H. Chan School of Public Health, Boston, Massachusetts; 5 RAND Corporation, Los Angeles, California; 6 University of Texas School of Public Health, Houston; 7 University of Alabama Birmingham, Birmingham; 8 University of Colorado, Aurora; 9 Northwestern University, Evanston, Illinois; 10 Kaiser Permanente Bernard J. Tyson School of Medicine, Pasadena, California

## Abstract

**Importance:**

In the United States, adolescents who are lesbian, gay, or bisexual (LGB) face disparities across physical and mental health outcomes compared with non-LGB youth, yet few studies have looked at patterns of health care utilization by sexual orientation.

**Objective:**

To compare health care utilization indicators for LGB and non-LGB youth.

**Design, Setting, and Participants:**

This cohort study analyzed wave 3 data from Healthy Passages, a longitudinal observational study of diverse public school students in Birmingham, Alabama; Houston, Texas; and Los Angeles County, California. Multivariable logistic regression models tested sexual-orientation differences in the past 12-month health care utilization measures, controlling for youth age, gender, race and ethnicity, household education, income, and marital status. Data collection began in 2010 when students were in the 5th grade (mean [SE] age, 11.13 [0.01] years) (wave 1) and continued 2 years later (wave 2, 7th grade) and 5 years later (wave 3, 10th grade). Permission to be contacted was provided for 6663 children, and 5147 (77%) participated in audio computer-assisted self-administered interviews. This study included 4256 youth (640 LGB, 3616 non-LGB) who completed interviews at wave 1 and wave 3 and answered key items used in this analysis. Analyses were completed in June 2021.

**Exposures:**

Sexual orientation (LGB vs non-LGB).

**Main Outcomes and Measures:**

Health care utilization and communication difficulty with a physician in the past 12 months.

**Results:**

Among 4256 youths included in the study at baseline in 5th grade (wave 1), 2171 (48.9%) were female; 1502 (44.5%) were Hispanic or Latino; 1479 (28.9%) were Black; the mean (SE) age was 11.19 (0.03) years; and 640 (14.5%) were LGB at wave 3. Compared with non-LGB youth, a higher proportion of LGB youth reported not receiving needed medical care in the last 12 months (adjusted odds ratio [aOR], 1.68; 95% CI,1.38-2.05), most commonly for sexually transmitted infections, contraception, and substance use. LGB youth more frequently reported difficulty communicating with their physician (aOR, 1.71; 95% CI, 1.27-2.30) than non-LGB youth.

**Conclusions and Relevance:**

This study’s results found that health care utilization differs by sexual orientation for youth. These findings suggest that clinician training is needed to address the health care needs of LGB youth. Routinely capturing sexual orientation data might enable tracking of health care utilization indicators for LGB youth.

## Key Points

**Question:**

Is reported health care utilization different for lesbian, gay, or bisexual (LGB) vs non-LGB youth?

**Findings:**

In this cohort study of 4256 diverse 10th graders randomly drawn from public schools in 3 geographic regions, LGB youth disproportionately reported not receiving needed medical care in the past 12 months relative to non-LGB youth.

**Meaning:**

This study’s findings suggest that health care utilization differs by sexual orientation for youth requiring interventions in clinical care settings.

## Teaser

This cohort study examines health care utilization indicators for lesbian, gay, and bisexual (LGB) youth and non-LGB youth.

## Introduction

In the US, lesbian, gay, and bisexual (LGB) people (also referred to as sexual minorities) face substantial disparities in physical and mental health outcomes.^1^ These disparities emerge in adolescence and young adulthood,^2,3^ including in mental health,^4,5^ substance use behaviors,^6,7,8^ HIV and other sexually transmitted infections (STIs),^9^ unintended teen pregnancy,^10,11^ cancer-related risk behaviors,^7,12^ preventive health behaviors,^13^ cardiovascular risk biomarkers,^14^ eating disorder behaviors,^15,16,17^ and chronic pain.^18^ Whether being a sexual minority is measured by sexual identity (self-identification as lesbian, gay, or bisexual), sexual attraction (attraction to people of the same and/or different gender), sexual behavior (same and/or different gender sexual contact), or some combination of all 3 dimensions, health disparities are ubiquitous for LGB youth.^19^

Health care utilization patterns, such as avoidance of or delay in seeking care, reduced access to health care, and difficulties communicating with clinicians, contribute to inequitable US health care outcomes.^20,21^ Despite LGB youth facing elevated population-health risks, few studies examine factors associated with their health care utilization.^1^ LGB adult research indicates substantial unmet medical needs, including needed care and preventive care.^22^ Reasons for not receiving needed care include reluctance to disclose sexual identity to clinicians, lower health insurance rates, lack of culturally appropriate preventive services, and lack of clinician LGB care competence.^23,24^ Among LGB youth, studies focused on clinician communication and clinician characteristics have identified confidentiality, medical knowledge, good communication skills, and mutual respect as most important.^25,26^ However, there is a gap in research concerning whether LGB youth, like LGB adults, avoid or delay seeking care. Understanding LGB youth’s health care utilization will inform interventions to improve their access and care.

The current study examined sexual-orientation differences in health care utilization in a large, diverse sample of 10th-grade public school students. Minority stress theory attributes sexual minority youths’ greater health risks and worse health outcomes to social stressors resulting from being in a stigmatized sexual minority group.^27^ LGB adolescents may anticipate adverse health care interactions owing to prior personal negative experiences, negative experiences of acquaintances who share their identity, and awareness of negative stereotypes regarding their identity. We therefore hypothesized that a lower proportion of LGB than non-LGB youth would be up-to-date in routine care and a higher proportion would report not receiving needed health care. We also anticipated that LGB youth would report greater difficulty communicating with a clinician and less comfort discussing sexual attractions with a clinician than non-LGB youth.

## Methods

### Study Overview and Procedures

This cohort study used data from Healthy Passages, a longitudinal study of diverse public school students from a stratified random sample of schools from Birmingham, Alabama; Houston, Texas; and Los Angeles County, California.^28,29^ Data collection began in 2010 when students were in the 5th grade (mean [SD] age, 11.1 [0.5] years) (wave 1) and continued 2 years later (wave 2, when most were in 7th grade) and 5 years later (wave 3, 10th grade). Permission to be contacted was provided for 6663 children; 5147 (77%) participated. Data were collected from children and parents via audio computer-assisted self-administered interviews in English or Spanish. Study methods and procedures are detailed elsewhere.^28^ All participants completed written procedures to obtain appropriate consent to participate. For the current study, wave 3 data were analyzed cross-sectionally and completed in June 2021. The inclusion criteria were respondents who completed the sexual orientation items. The analytic sample and denominator for this study was 4256 youths (640 LGB, 3616 non-LGB). These youth completed interviews at wave 1 and wave 3 and answered key items used in this analysis. Study activities were reviewed and approved by the institutional review boards of each participating study site. This study followed the Strengthening the Reporting of Observational Studies in Epidemiology (STROBE) reporting guideline.

### Measures

#### Sociodemographics (Covariates)

Parent-reported sociodemographics were from wave 1. Sociodemographics included child age in years, child gender (female or male; the survey did not ask about having a transgender child), child race and ethnicity, highest level of education in household, household income, and marital status.

#### Sexual Minority Status (Primary Independent Variable)

Sexual minority status (LGB vs non-LGB) was derived from the combined responses to 2 self-reported items in the 10th-grade youth survey that are similar to items used in other studies^30^: (1) “People differ in their sexual attraction to other people. Which best describes your feelings?” (Response options: “Attracted only to girls,” “Attracted mostly to girls and a little to boys,” “Attracted equally to girls and boys,” “Attracted mostly to boys and a little to girls,” “Attracted only to boys,” and “You are not sure”) and (2) “Which best describes you?” (Response options: “100% heterosexual/straight,” “Mostly heterosexual/straight,” “Bisexual,” “Mostly homosexual/gay/lesbian,” “100% homosexual/gay/lesbian,” and “You are not sure”). Youth were defined as being a sexual minority if they reported at least a little attraction to the same sex or not being 100% heterosexual/straight.^31,32,33^

#### Health Care Utilization Outcomes

Two indicators were operationalized for health care utilization: (1) routine care: youth were asked the last time they went to a physician or other health care clinician for regular or routine care such as a regular checkup when they were not sick; and (2) other care: youth were asked the last time they went to a physician or other health care clinician for anything else (eg, when they were sick, not a visit for a routine check-up or physical exam). Response options for both questions were: 1 indicating 0 to 6 months; 2 indicating 7 to 12 months; 3 indicating 13 to 24 months; 4 indicating more than 2 years ago. Responses were dichotomized to capture not receiving care in the past 12 (or 24) months.

#### Parent-Reported Delayed Care and Youth Self-reported Serious Problem That Went Untreated

Parents reported whether in the last 12 months they delayed or went without needed health care for their child (yes or no). Youth self-reported whether they had a serious health problem that went untreated in the last 12 months (yes or no). Parent-reported delaying care and youth-reported untreated health problem had very low levels of agreement (κ = 0.06; 95% CI, 0.02-0.09).

#### Unmet Medical Care Needs, Type of Medical Care Needed, and Reasons for Not Seeking Care Outcomes

Youth were asked whether there was a time in the past 12 months when they thought they should get medical care (eg, a regular check-up, visit for illness, or a visit for another reason) but they did not. A series of follow-up questions using a check-all-that-apply format queried youth about the type of care they think they needed (eg, regular check-up, visit for STIs, visit for contraception), and their reasons for not seeing a health care clinician (eg, did not know who to see, did not want parents to know, difficult to make an appointment).

#### Communication With Health Care Clinician Outcomes

Youth were asked whether there was a time, in the past 12 months, when they saw a physician or health care clinician and wanted to talk about a topic or problem with him or her but were not able to (yes or no). Follow-up prompts assessed reasons for not communicating with a clinician in a check-all-that-apply format (eg, health care clinician did not bring up the topic, did not want parents to know, afraid of what the physician would say or do). Youth were asked how comfortable they feel talking to their physician or health care clinician about whether they are attracted to boys, girls, or both. Likert response options were 1 indicating very comfortable; 2 indicating somewhat comfortable; 3 indicating a little comfortable; 4 indicating not at all comfortable, coded dichotomously to compare very or somewhat with a little or not at all comfortable. Only self-identified LGB youth were asked whether any physician or other health care clinician knows they are bisexual, mostly homosexual, or 100% homosexual, gay, or lesbian (yes or no).

### Statistical Analysis

Cross-sectional descriptive statistics characterized the overall sample of youth by sexual minority status (LGB vs non-LGB). Bivariate analyses assessed whether the distribution of key demographic variables differed by LGB status. Separate logistic regression models were fit regressing each sociodemographic variable on LGB status, accounting for complex survey design (SAS proc survey–weights, cluster, strata). Odds ratios (OR) and 95% CIs were estimated for bivariate models. Variables statistically significant at the *P* < .05 level in bivariate models and conceptualized as confounders were retained in multivariable regression models.

Separate multivariable logistic regression models were fit regressing each outcome on sexual minority status (LGB vs non-LGB youth), and controlling for student wave 1 age in years, gender, race and ethnicity, and household education, income, and marital status. A logistic regression model restricted to LGB youth examined whether comfort level talking to a clinician about sexual orientation was associated with any physician or health care clinician knowing youths’ sexual minority status. Adjusted ORs (aORs) and 95% CIs for multivariable logistic regression models are presented. All statistical analyses were adjusted for the complex sampling and survey design (weight, cluster, strata). Covariate-adjusted proportions were obtained using a recycled predictions approach. Complete case analyses were conducted because missing data percentages were low (<10%) and did not significantly differ for LGB vs non-LGB youth. Per the STROBE guidelines,^34^ a sensitivity analysis was conducted using multiple imputation procedures to handle missing data values and found similar results to the complete case analyses in terms of direction, magnitude, and significance.

Statistical analysis was performed using SAS statistical software version 9.4 (SAS Institute) from June 2020 to July 2021. Statistical tests were 2-tailed and significance was set at *P* < .05.

## Results

### Descriptive Characteristics

Among 4256 youths included in the study at baseline in 5th grade (wave 1), 2171 (48.9%) were female, 1502 (44.5%) were Hispanic or Latino, and 1479 (28.9%) were Black; the mean (SE) age was 11.13 (0.01) years; 640 (14.5%) were LGB at wave 3. Table 1 presents sample sociodemographics by sexual orientation. In bivariate comparisons with non-LGB youth, LGB youth were on average older at wave 1 in households with lower educational attainment and income, less likely to be White, and more likely to be female and with unmarried parents. Household employment and health insurance did not differ significantly by sexual orientation.

**Table 1.** Sociodemographic Characteristics by Sexual Orientation for LGB and Non-LGB Youth^a^

Characteristic	No. (%)^b^			Bivariate comparisons: LGB vs non-LGB, OR (95% CI)^c^
	LGB	Non-LGB	Total sample	
Total	640 (14.5)	3616 (85.5)	4256 (100.0)	NA
Age at wave 1, mean (SE), y (n = 4256)	11.19 (0.03)	11.12 (0.01)	11.13 (0.01)	β, 0.07 (0.01-0.13)
Gender (n = 4256)				
Male	175 (28.0)	1910 (55.0)	2085 (51.1)	1 [Reference]
Female	465 (72.0)	1706 (45.0)	2171 (48.9)	3.14 (2.50-3.94)
Race and ethnicity (n = 4255)				
Black	237 (31.9)	1242 (28.5)	1479 (28.9)	1.64 (1.27-2.13)
Hispanic	244 (47.2)	1258 (43.0)	1502 (44.5)	1.57 (1.23-2.01)
White	118 (15.8)	910 (23.2)	1028 (22.1)	1 [Reference]
Other	40 (5.1)	206 (4.3)	246 (4.5)	1.73 (1.05-2.86)
Highest level of education in household (n = 4197)				
Some high school without graduation	124 (27.0)	626 (23.0)	750 (23.6)	1.69 (1.29-2.22)
High school graduate or GED	124 (21.5)	721 (21.9)	845 (21.9)	1.41 (1.05-1.89)
Some college or 2-y degree	208 (30.2)	940 (24.3)	1148 (25.1)	1.79 (1.42-2.27)
4-y college graduate or more	171 (21.3)	1283 (30.8)	1454 (29.4)	1 [Reference]
Household (n = 4205)				
Employment				
Parent or guardian employed full-time	341 (50.9)	2026 (54.5)	2367 (54.0)	0.86 (0.72-1.04)
Parent or guardian not employed full-time	288 (49.1)	1550 (45.5)	1838 (46.0)	1 [Reference]
Marital status (n = 4250)				
Not married	356 (53.8)	2213 (63.9)	2569 (62.4)	1.52 (1.22-1.90)
Married	284 (46.2)	1397 (36.1)	1681 (37.6)	1 [Reference]
Income, $ (n = 4256)				
<30 000 (<median)	287 (48.7)	1411 (42.2)	1698 (43.1)	1.35 (1.12-1.64)
≥30 000 (>median)	306 (42.1)	1955 (49.2)	2261 (48.2)	1 [Reference]
Missing	47 (9.2)	250 (8.6)	297 (8.7)	1.24 (0.87-1.76)
Health insurance (n = 4254)				
No	68 (11.6)	341 (10.8)	409 (10.9)	1.08 (0.82-1.42)
Yes	571 (88.4)	3274 (89.2)	3845 (89.1)	1 [Reference]

Abbreviations: GED, general education diploma; LGB, lesbian, gay, bisexual, or other sexual minority youth; NA, not applicable; non-LGB, heterosexual or straight youth; OR, odds ratio.

^a^ All sociodemographics are from wave 1 when students were in 5th grade. Sexual orientation is from wave 3 in 10th grade.

^b^ Weighted % and unweighted No. are presented.

^c^ Bivariate comparisons by LGB status are adjusted for complex sampling and survey design.

### Health Care Utilization

Table 2 presents health care utilization behaviors among youth by sexual orientation in 10th grade. Past 12-month and past 24-month routine care and other care access did not significantly differ for LGB vs non-LGB youth.

**Table 2.** Health Care Utilization Behaviors of Youth by Sexual Orientation in 10th Grade^a^

Variable	LGB		Non-LGB		Total sample		Multivariable models: LGB vs non-LGB, aOR (95% CI)
	% (95% CI)	No.	% (95% CI)	No.	% (95% CI)	No.	
No routine care: did not go to a physician or other health care clinician for regular or routine care (eg, when you were not sick)							
No.	4233						
Not in the past 12 mo	13.9 (13.8-13.9)	79	10.6 (10.5-10.6)	372	11.8 (11.8-11.9)	451	1.35 (0.95-1.92)
Not in the past 24 mo	6.2 (6.1-6.3)	33	4.2 (4.1,4.2)	147	4.9 (4.9-5.0)	180	1.49 (0.90-2.50)
No other care: did not go to a physician or other health care clinician for anything else (eg, when you were sick)							
No.	4236						
Not in past 12 mo	17.6 (17.4-17.7)	94	14.3 (14.2-14.4)	521	15.5 (15.5-15.6)	615	1.28 (0.92-1.78)
Not in past 24 mo	8.5 (8.4-8.6)	45	7.2 (7.2-7.3)	271	7.7 (7.7-7.8)	316	1.19 (0.73-1.94)

Abbreviations: aOR, adjusted odds ratio; LGB, lesbian, gay, bisexual, and other sexual minority youth; non-LGB, heterosexual or straight youth.

^a^ Weighted percentage and unweighted number are presented. Covariate-adjusted proportions are presented which were derived using a recycled predictions approach. Separate multivariable regression models appropriately adjusted for survey methodology (proc survey with weights, cluster, and strata) were fit regressing each health care indicator on LGB status controlling for youth age in years, youth gender, youth race and ethnicity, household education, household income, and marital status.

### Parent-Reported Delaying Needed Care for Child and Youth-Reporting a Serious Health Problem That Went Untreated

There were no significant sexual orientation differences in parent-reported delaying their child’s needed health care. However, a significantly higher proportion of LGB than non-LGB youth reported having a serious problem that went untreated in the past 12 months (8.3% [95% CI, 8.2%-8.4%] vs 3.7% [95% CI, 3.7%-3.7%]; aOR, 2.36 [95% CI, 1.65-3.37]) (Table 3).

**Table 3.** Parent-Reported Delay in Needed Care and Youth-Reported Unmet Medical Care Needs: Type of Medical Care Needed and Reasons for Not Seeking Care by Sexual Orientation in 10th Graders^a^

Variable	LGB		Non-LGB		Total sample		Multivariable models: LGB vs non-LGB, aOR (95% CI)
	% (95% CI)	No.	% (95% CI)	No.	% (95% CI)	No.	
Parent-reported delayed needed care: parent reported delaying needed health care for child^b^							
No.	4252						
During the past 12 mo	10.9 (10.8-11.0)	70	9.7 (9.6-9.8)	349	10.1 (10.1-10.2)	419	1.13 (0.84-1.51)
Serious health problem went untreated: had a serious health problem that went untreated							
No.	4238						
During the past 12 mo	8.3 (8.2-8.4)	58	3.7 (3.7-3.7)	136	5.5 (5.4-5.5)	194	2.36 (1.65-3.37)
Did not get medical care when needed: thought you should get medical care (eg, a regular checkup visit for illness or a visit for another reason) but you did not?							
No.	4233						
During the past 12 mo	42.4 (42.2-42.6)	277	30.2 (30.1-30.4)	1081	34.9 (34.7-35.0)	1358	1.68 (1.38-2.05)
Type of medical care needed: what type of medical care did you think you needed?							
Regular checkup or routine physical exam	57.3 (56.9-57.6)	175	67.6 (67.3-67.8)	728	63.6 (63.4-63.9)	903	0.61 (0.45-0.83)
Visit for illness other than for STIs	23.1 (22.8-23.4)	63	21.4 (21.2-21.6)	226	22.1 (21.9-22.2)	289	1.12 (0.76-1.66)
Visit for STIs	10.2 (10.1-10.3)	33	5.5 (5.5-5.6)	60	7.3 (7.2-7.4)	93	1.92 (1.15-3.19)
Visit for contraception	3.9 (3.8-4.0)	17	1.2 (1.2-1.3)	18	2.3 (2.2-2.3)	35	3.00 (1.29-6.94)
Visit for substance use	5.1 (4.8-5.3)	7	1.1 (1.1-1.2)	11	2.6 (2.5-2.7)	18	5.25 (2.08-13.29)
Other type of visit	28.6 (28.4-28.9)	84	23.7 (23.5-23.9)	257	25.6 (25.4-25.7)	341	1.29 (0.88-1.89)
Reasons for not seeking medical care: what kept you from seeing a health clinician when you really needed to?							
Did not know whom to see	15.3 (15.1-15.4)	38	17.2 (17.1-17.4)	181	16.5 (16.4,16.6)	219	0.87 (0.55-1.35)
Had no transportation	19.9 (19.7-20.0)	59	14.7 (14.6-14.8)	156	16.6 (16.5-16.8)	215	1.42 (0.97-2.07)
No one available to go along	7.7 (7.6-7.9)	20	5.5 (5.5-5.6)	59	6.4 (6.3-6.5)	79	1.40 (0.76-2.58)
Parent or guardian would not go	12.7 (12.5-12.9)	41	9.4 (9.2-9.5)	98	10.6 (10.5-10.7)	139	1.40 (0.87-2.25)
Did not want parents to know	14.5 (14.4-14.7)	48	7.1 (7.0-7.2)	78	9.9 (9.8-10.0)	126	2.22 (1.46-3.38)
Afraid the visit would not be confidential	3.9 (3.8-4.0)	15	2.8 (2.7-2.8)	28	3.2 (3.1-3.2)	43	1.38 (0.69-2.75)
Difficult to make appointment	8.6 (8.5-8.7)	29	13.0 (12.9-13.1)	130	11.3 (11.2-11.4)	159	0.62 (0.39-0.99)
Afraid of what physician would say or do	7.6 (7.5-7.7)	27	4.8 (4.7-4.8)	51	5.8 (5.8-5.9)	78	1.59 (0.90-2.82)
Thought the problem would go away	27.8 (27.4-28.2)	77	26.3 (26.0-26.6)	270	26.8 (26.6-27.0)	347	1.11 (0.78-1.59)
Could not pay for a visit	9.8 (9.7-10.0)	30	7.0 (6.9-7.1)	68	8.1 (8.0-8.2)	98	1.42 (0.81-2.49)
Other	25.2 (25.1-25.3)	60	21.5 (21.5-21.6)	228	22.9 (22.9-23.0)	288	1.22 (0.84-1.75)

Abbreviations: aOR, adjusted odds ratio; LGB, lesbian, gay, bisexual, and other sexual minority youth; non-LGB, heterosexual or straight youth; STIs, sexually transmitted infections.

^a^ Weighted percentage and unweighted number are presented. Covariate-adjusted proportions are presented which were derived using a recycled predictions approach. Separate multivariable logistic regression models adjusting for complex survey design (proc survey with weights, cluster, and strata) were fit regressing each health care indicator on LGB status controlling for child age in years, youth gender, youth race and ethnicity, household education, household income, and marital status.

^b^ Parent-reported delayed needed care variable; all other variables were self-reported by youth.

### Unmet Medical Care Needs, Type of Medical Care Needed, and Reasons for Not Seeking Care

A higher proportion of LGB than non-LGB youth did not get needed medical care in the last 12 months, including a regular check-up, visit for illness, or visit for another reason (42.4% [95% CI, 42.2%-42.6%] vs 30.2% [95% CI, 30.1%-30.4%]; aOR, 1.68 [95% CI, 1.38-2.05]). Medical care needed for LGB vs non-LGB youth was disproportionately for STIs (10.2% [95% CI, 10.1%-10.3%] vs 5.5% [95% CI, 5.5%-5.6%]), contraception (3.9% [95% CI, 3.8%-4.0%] vs 1.2% [95% CI, 1.2%-1.3%]), and substance use (5.1% [95% CI, 4.8%-5.3%] vs 1.1% [95% CI, 1.1%-1.2%]). Not wanting their parents to know was more often a reason why LGB vs non-LGB youth did not seek needed care (14.5% [95% CI, 14.4%-14.7%] vs 7.1% [95% CI, 7.0%-7.2%]; aOR, 2.22 [95% CI, 1.46-3.38]); difficulty making an appointment was less often a reason for sexual minority youth than heterosexual youth (8.6% [95% CI, 8.5%-8.7%] vs 13.0% [95% CI, 12.9%-13.1%]; aOR, 0.62 [95% CI, 0.39-0.99]). LGB youth were less likely than non-LGB youth to have unmet need for a regular check-up or routine physical exam (57.3% [95% CI, 56.9%-57.6%] vs 67.6% [95% CI, 67.3%-67.8%]).

### Communication With a Health Care Clinician

LGB youth were more likely than non-LGB youth to report not communicating with a health care clinician about a topic or problem needing discussion (15.3% [95% CI, 15.2%-15.4%] vs 9.4% [95% CI, 9.3%-9.4%]; aOR, 1.71 [95% CI, 1.27-2.30]). Commonly reported reasons for youth not communicating were: not wanting parents to know, embarrassment to talk about the topic, and health care clinician not bringing up the topic. There were no significant sexual-orientation differences in reasons youth endorsed for not communicating (Table 4).

**Table 4.** Communication With a Healthcare Clinician by Sexual Orientation in 10th Graders^a^

Variable	LGB		Non-LGB		Total sample		Multivariable Models: LGB vs non-LGB, aOR (95% CI)
	% (95% CI)	No.	% (95% CI)	No.	% (95% CI)	No.	
No communication with clinician about topic or problem needing discussion: is there a time when you saw a physician or health care clinician and wanted to talk about a topic or problem with him or her but weren’t able to?							
No.	3948						
During the past 12 mo	15.3 (15.2-15.4)	103	9.4 (9.3-9.4)	325	11.6 (11.6-11.7)	428	1.71 (1.27-2.30)
Reasons did not communicate: what kept you from talking to the health professional about the topic or issue?							
Health care clinician did not bring up the topic	26.9 (26.6-27.2)	25	27.1 (26.9-27.3)	74	27.0 (26.9-27.2)	99	0.99 (0.38-2.28)
Health care clinician did not allow you to ask	6.1 (5.9-6.3)	7	8.2 (8.0-8.4)	21	7.4 (7.3-7.5)	28	0.74 (0.25-2.22)
No time; visit was too rushed	18.3 (18.0-18.5)	18	21.3 (21.1-21.5)	64	20.1 (20.0-20.3)	82	0.83 (0.42-1.62)
Did not want parents to know	40.7 (40.1-41.2)	43	31.8 (31.4-32.3)	111	35.2 (34.9-35.5)	154	1.50 (0.77-2.90)
Afraid the visit would not be confidential	15.2 (15.0-15.5)	19	11.4 (11.2-11.6)	39	12.9 (12.7-13.0)	58	1.39 (0.69-2.83)
Afraid of what the physician would say or do	14.3 (14.1-14.6)	18	13.1 (13.0-13.3)	40	13.6 (13.5-13.8)	58	1.10 (0.48-2.53)
Thought the issue would go away	20.7 (20.4-20.9)	20	16.6 (16.4-16.7)	50	18.1 (18.0-18.3)	70	1.33 (0.68-2.61)
Embarrassed to talk about the topic or issue	37.5 (37.1-37.9)	41	25.9 (25.6-26.2)	90	30.3 (30.0-30.6)	131	1.72 (0.91-3.26)
Other	8.2 (8.0-8.4)	8	10.2 (10.0-10.3)	32	9.4 (9.3-9.5)	40	0.79 (0.29-2.14)
Comfort level talking about sexual orientation with physician or health care clinician: how comfortable do you feel talking to your physician or health care clinician about whether you are attracted to boys, girls, or both?							
No.	4241						
Very or somewhat comfortable	34.2 (34.0-34.5)	199	62.2 (62.0-62.4)	2309	51.5 (51.2-51.8)	2508	0.31 (0.25-0.39)
A little or not at all	65.8 (65.5-66.0)	437	37.8 (37.6-38.0)	1296	48.5 (48.2-48.8)	1733	1 [Reference]

Abbreviations: aOR, adjusted odds ratio; LGB, lesbian, gay, bisexual, and other sexual minority youth; non-LGB, heterosexual or straight youth.

^a^ Weighted percentage and unweighted number are presented. Covariate-adjusted proportions are presented which were derived using a recycled predictions approach. Separate multivariable logistic regression models adjusting for complex survey design (proc survey with weights, cluster, and strata) were fit regressing each health care indicator on LGB status controlling for youth age in years, youth gender, youth race and ethnicity, household education, household income, and marital status.

A significantly lower proportion of LGB than non-LGB youth felt very or somewhat comfortable talking to their health care clinician about whether they are attracted to boys, girls, or both (34.2% [95% CI, 34.0%-34.5%] vs 62.2% [95% CI, 62.0%-62.4%]; aOR, 0.31 [95% CI, 0.25-0.39]). Most LGB youth (65.8% [95% CI, 65.5%-66.0%]) reported feeling a little or not at all comfortable talking to a health care clinician about their sexual attractions, compared with only 37.8% (95% CI, 37.6%-38.0%) of non-LGB youth. Overall, only 12.5% (unweighted n = 20) of LGB youth reported that a physician or other health care clinician knew they were bisexual, mostly homosexual, or 100% homosexual, gay, or lesbian. LGB youth who were not at all comfortable talking to a physician or health care clinician about their sexual orientation were much less likely to report any health care clinician knowing that they are a sexual minority (7.7% [95% CI, 0.0%-19.8%] vs 47.1% [95% CI, 38.9%-55.2%]; OR, 0.07 [95% CI, 0.01-0.38]) (Table 4).

## Discussion

Healthy People 2020 identified eliminating sexual minorities’ health disparities, including increasing access to high-quality health care services, as a priority for research and interventions.^35^ In this study, reported health care use differed by sexual orientation: LGB youth more often had unmet treatment needs. Minority stress theory^27^ postulates that health care utilization for LGB youth is likely influenced by stigma. LGB youth were less likely to seek needed care and raise health concerns during a visit. Parent reports of delaying needed care for their child did not differ by youth LGB status; however, LGB youth more often reported having a serious health problem that went untreated in the prior 12 months. The lack of agreement in parent-child reports highlights the need to gather both parent and child perspectives in understanding health care utilization. These findings support the need to ask youth directly about their needs and to address unmet needs of LGB youth. This study adds to the limited health services research characterizing adolescent health care utilization by sexual orientation.

Contrary to our hypothesis, LGB youth were less likely than non-LGB youth to endorse a regular check-up or routine physical exam as an unmet medical need. LGB youth may not believe they need routine care or may not discuss their specific needs during regular check-ups. However, we found support for the hypothesis that LGB youth were more likely than non-LGB youth to have unmet care needs in acute care or problem-based health needs for a specific issue. Unmet medical care needs for LGB youth were more often STIs, contraception, and substance use, areas where pronounced health disparities exist for LGB populations.^9,11,12^ LGB health disparities may be exacerbated by not receiving needed care in these health domains and subsequent delays, or absence, of treatment.

LGB youth may have increased vigilance and anxiety surrounding sexual health due to fears of confirming negative stereotypes about their group when seeking health care for these issues. This so-called stereotype threat may arise where individuals are concerned about being judged or treated stereotypically, or conforming to a group stereotype.^36^ Such a threat may cause LGB-identified youth to avoid health care settings or clinician conversations on these topics.^37^ For example, a gay male youth may not want to bring up STI screening with his clinician because he does not want to invoke the stereotype that all gay men are at risk for STIs. Research would benefit from investigating this further to inform patient-clinician interventions to overcome barriers to care.

LGB adolescents were more likely than non-LGB counterparts to endorse did not want parents to know and less likely to report difficulty making an appointment as a reason for not seeing a clinician for needed care. Thus, issues of family engagement, rather than health care navigation issues, are associated with avoidance behaviors for LGB youth. Most states have some form of minor consent laws pertaining to seeking sexual health care (eg, STI treatment, contraception) without parental notification or consent. Clinicians should familiarize themselves with relevant laws to know when parental notification is required.^35,38,39,40^ Clinicians should also reassure their adolescent patients by explicitly stating the confidentiality of their encounters.^30^

As anticipated, LGB youth self-reported greater difficulty communicating with a clinician about topics they wanted to discuss. However, there were no statistically significant sexual-orientation differences in reasons why the communication difficulty was present. Most (65.8%) LGB youth reported feeling a little or not at all comfortable talking to a health care clinician about their sexual attractions, compared with only 37.8% of non-LGB youth. Consistent with prior disclosure research,^41^ only 12.5% of LGB adolescents here indicated that a clinician knew they were LGB. Health care clinicians must be aware of youths’ sexual orientation to provide high-quality, comprehensive care. This is particularly relevant in the context of the negative health consequences of LGB internalized stigma, and the pertinence of certain sexual behaviors to determine appropriate preventive care and screening within this population (eg, depression screening, STI screening, pre-HIV-exposure prophylaxis, safe sexual practices).

The American Medical Association, the American Academy of Pediatrics, and the Society for Adolescent Health and Medicine recommend discussing sexual orientation as part of the health care of all adolescents.^42,43,44,45,46,47,48^ Given the power dynamic of a patient-clinician interaction and the perceived risk of stigmatization during the medical encounter, adolescents may not feel comfortable discussing their sexual orientation with a clinician. Because of this, it is essential that pediatricians have the knowledge and skills needed to initiate these conversations and establish a trusting relationship with their adolescent patients to provide them with needed care.

### Limitations

This study’s findings should be interpreted alongside several limitations. First, this is a cohort study and cross-sectional analysis where associations between variables are not causal. Additional research is needed to establish the mechanisms behind these sexual-orientation differences. Also, studies examining the measurement of sexual orientation among adolescents have shown that different dimensions of sexual orientation (attraction, behavior, identity) can be incongruent, leading any method of sexual orientation operationalization to have certain weaknesses.^40^ We used a composite of 2 dimensions, sexual identity and sexual attraction, to better capture sexual orientation. We also chose this measure to be able to compare these findings with previous research. This method did not take into account youth uncertainty about their sexual orientation. The demographic and geographic diversity of our sample is a strength, but results may not be generalizable to youth outside of the contexts from which participants were drawn. Furthermore, the study, conducted in English and Spanish, may underrepresent youth who prefer other languages. Parent-reported sociodemographic data occurred in wave 1, so it may have changed by wave 3 when youth outcomes were assessed. Additionally, disclosure of sexual orientation to parents may play a role in youth’s access to and utilization of care and should be considered in future studies. This study did not include information about gender identity, so we were not able to comment on the needs of transgender youth.

## Conclusions

This study found that LGB youth reported more unmet treatment needs, less seeking of needed care, and more difficulty communicating concerns to clinicians than non-LGB peers. Health care that is responsive to the needs and concerns of LGB youth and concomitant health services research is needed to reduce health disparities seen in this population. Care should be sensitive and respectful to sexual orientation for all youth, with clinicians taking time to ask adolescents about their sexual identity, attractions, and behaviors, particularly in sexual and reproductive health.
